# Identification and validation of the pyroptosis-related long noncoding rna signature to predict the prognosis of patients with bladder cancer

**DOI:** 10.1097/MD.0000000000033075

**Published:** 2023-02-22

**Authors:** Zhenyu Wu, Jie Zeng, Mengxi Wu, Quan Liang, Bin Li, Guoliang Hou, Zhe Lin, Wenfeng Xu

**Affiliations:** a Department of Urology, The First People’s Hospital of Foshan, Foshan, Guangdong, China; b Department of Thoracic Surgery, Guangzhou First People’s Hospital, South China University of Technology, Guangzhou, Guangdong, China; c Department of Thoracic Surgery, Shenzhen Hospital, Southern Medical University, Shenzhen, Guangdong, China.

**Keywords:** bladder cancer, lncRNA, prognostic model, pyroptosis

## Abstract

Bladder cancer ranked the second most frequent tumor among urological malignancies. This work investigated bladder cancer prognosis, including the relevance of pyroptosis-related long noncoding RNA (lncRNA) in it and its potential roles. The Cancer Genome Atlas database offered statistics on lncRNAs and clinical data from 411 bladder cancer patients. Pearson correlation analysis was used to evaluate pyroptosis-related lncRNAs. To explore prognosis-associated lncRNAs, we performed univariate Cox regression, least absolute shrinkage and selection operator regression analyses, as well as the Kaplan–Meier method. Multivariate Cox analysis was leveraged to establish the risk score model. Afterward, a nomogram was constructed according to the risk score and clinical variables. Finally, to investigate the potential functions of pyroptosis-related lncRNAs, gene set enrichment analysis was employed. Eleven pyroptosis-related lncRNAs were screened to be closely associated with patients prognosis. On this foundation, a risk score model was created to classify patients into high and low risk groups. The signature was shown to be an independent prognostic factor (*P* < .001) with an area under the curve of 0.730. Then a nomogram was established including risk scores and clinical characteristics. The nomogram prediction effect is excellent, with a concordance index of 0.86. The 11-lncRNAs signature was associated with the supervision of oxidative stress, epithelial-mesenchymal transition, cell adhesion, TGF-β, and Wingless and INT-1 signaling pathway, according to the gene set enrichment analysis. Our findings indicate that pyroptosis-related lncRNAs, which may affect tumor pathogenesis in many ways, might be exploited to assess the prognosis of bladder cancer patients.

## 1. Introduction

As the world’s number 10 most frequent malignant tumor and number 2 most prevalent urological malignancy, bladder cancer has caused a total of 213,000 deaths and 573,000 new cases in 2020.^[1]^ Patients with bladder cancer receive a prognosis that is closely linked to the pathological diagnosis, comprising non muscle invasive bladder cancer as well as muscle invasive bladder cancer. Most non muscle invasive bladder cancer patients will eventually turn into muscle invasive bladder cancer, which has a 5-year survival rate of fewer than 50%.^[2,3]^ Prognosis prediction of bladder cancer is still a challenge for it may be associated with biological heterogeneity.^[4]^ Because current approaches are insufficient for reliably assessing the prognosis of bladder cancer patients, a more trustworthy method must be investigated.

Long noncoding RNAs (lncRNAs) play a significant part in a great variety of physiological and pathological functions, especially in tumor growth and progression.^[5,6]^ It was reported that lncRNAs played a role in tumorigenesis and metastasis through the regulation of cell proliferation, differentiation, and migration.^[7]^ Accumulating evidence suggested that lncRNAs may be a viable therapeutic target as well as a prognosis-predicting tumor marker.^[8,9]^ Although some lncRNAs have been successfully used to predict bladder cancer prognosis,^[10,11]^ the prognostic value of pyroptosis-related lncRNAs in bladder cancer remains to be studied.

Pyroptosis, a type of programmed cell death triggered by inflammasomes, was linked to cancer cell proliferation, invasion, and metastasis.^[12–14]^ In various malignancies, novel pyroptosis-related markers have been developed in recent investigations. Zhang et al^[15]^ discovered that 4 pyroptosis-related genes play a significant role in endometrial cancer prognosis. Ovarian cancer prognosis can also be predicted using pyroptosis-related genes.^[16]^ For bladder cancer, Chen et al^[17]^ found that the prognosis model established by pyroptosis-related genes can predict the patient prognosis. However, more research is needed into the potential functions and prognostic role of pyroptosis-related lncRNAs in bladder cancer.

In this study, pyroptosis-related lncRNAs were identified by analyzing the expression of lncRNAs and the related clinical data from the cancer genome atlas (TCGA). By dint of these lncRNAs, a prognostic model was established, and their possible biological functions in bladder cancer were also investigated.

## 2. Materials and methods

### 2.1. Pyroptosis-related lncRNAs identification

From TCGA (https://cancergenome.nih.gov/), we acquired bladder cancer patients lncRNA and clinical data. Based on previous publications, 52 pyroptosis-related genes were extracted.^[18–21]^ The criteria for identifying differentially expressed lncRNAs and pyroptosis-related genes between malignancies and normal adjacent tissues were log2FC  > 0.5 and *P* < .05. Pyroptosis-related lncRNAs were evaluated using pearson correlation analysis, with univariate cox regression also used for the exploration of prognosis-related lncRNAs. To recognize lncRNAs with prognostic value, the least absolute shrinkage and selection operator regression as well as Kaplan–Meier analyses were utilized.

### 2.2. Risk score model establishment

The coefficient (βi) was computed using the screened lncRNAs in a multivariate Cox regression model. A risk score model was subsequently built including the lncRNA expression levels and βi. This formula was used to compute each patient risk score. Furthermore, according to the median risk score, we classified patients into 2 categories: a high-risk group and a low risk group. Then, the Kaplan–Meier survival curve revealed the prognostic discrepancy between the 2 groups.

### 2.3. Nomogram construction and evaluation

We used univariate and multivariate Cox regression analyses in our investigation of the prognostic relevance of the risk score and clinical factors including gender, age, and the TMN stage. Next, using the findings of multivariate Cox regression, a nomogram was constructed for the prediction of each bladder cancer patient 3-year and 5-year overall survival (OS). Finally, the prediction accuracy of the nomogram was measured with the receiver operating characteristic curve analysis and the concordance index, and its prediction performance was assessed using calibration curves and decision curve analysis.

### 2.4. Gene set enrichment analysis

On these pyroptosis-related lncRNAs, the gene set enrichment analysis (GSEA) was carried out to not only apply the gene ontology enrichment analysis but also utilize the kyoto encyclopedia of genes and genomes pathway analysis, to further probe into their potential pathways and functions.

### 2.5. Ethics approval and consent to participate

All data of our research were derived from TCGA database and required no ethical approval.

### 2.6. Statistical analysis

Statistical analyses of this research were entirely conducted in the R software (version 4.0.2) (Institute for Statistics and Mathematics, Vienna, Austria; https://www.r-project.org). To identify the relationships between pyroptosis-related genes and lncRNAs, a Pearson correlation analysis was carried out. For comparison of the differences between categorical and continuous variables, the Chi-square test, as well as the *t* test were used, respectively. In evaluating the OS of patients in various groups, the Kaplan–Meier method was utilized, and differences between groups were assessed utilizing the log-rank test. Both univariate and multivariate Cox regression analyses were leveraged to carry out the survival analysis. At a 2-tailed *P* < .05, the results were regarded as statistically significant.

## 3. Results

### 3.1. Pyroptosis-related lncRNAs identification

**Figure 1.**
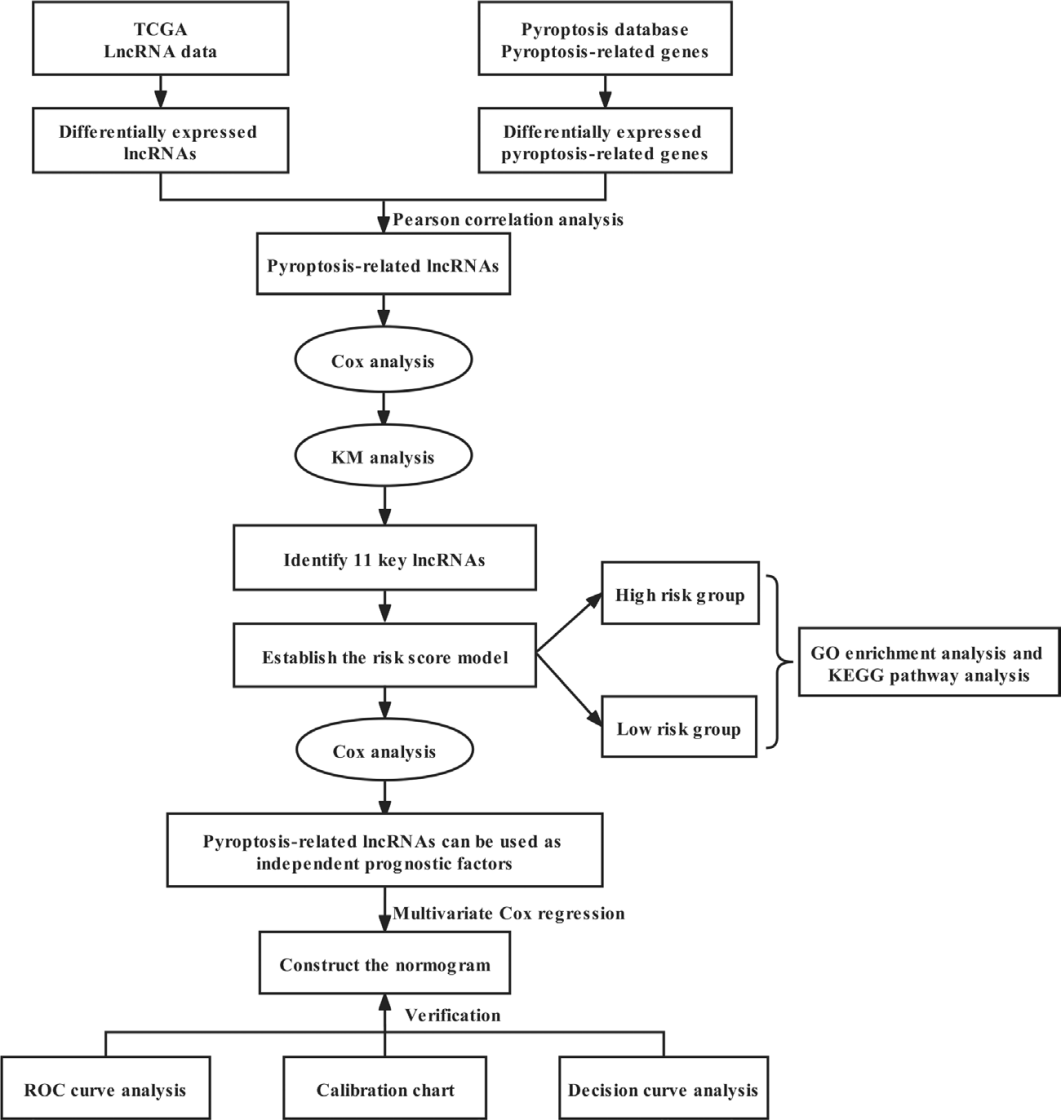
This study’s design and flowchart.

**Figure 2.**
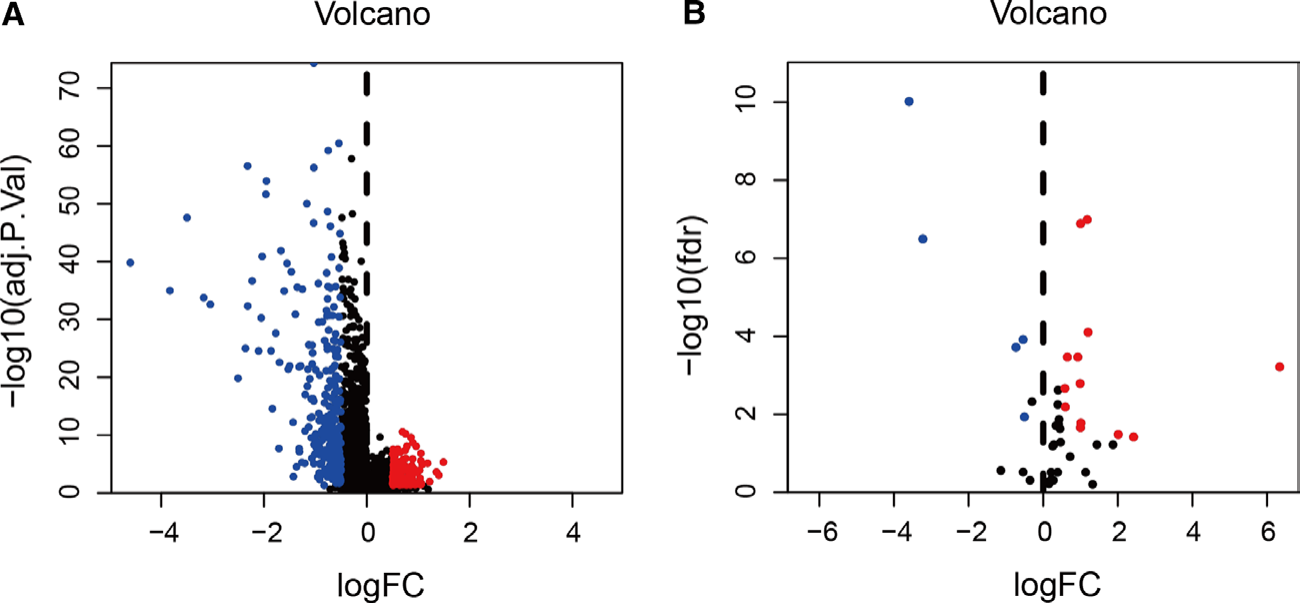
Identifying pyroptosis-related lncRNA in the tumor. (A) The volcano plot revealed that in tumors, 161 lncRNAs were up-regulated whereas 293 were down regulated. (B) The volcano plot visualized that in tumors, 7 pyroptosis-related genes were up-regulated and 8 were down regulated. lncRNA = long noncoding RNA.

**Figure 3.**
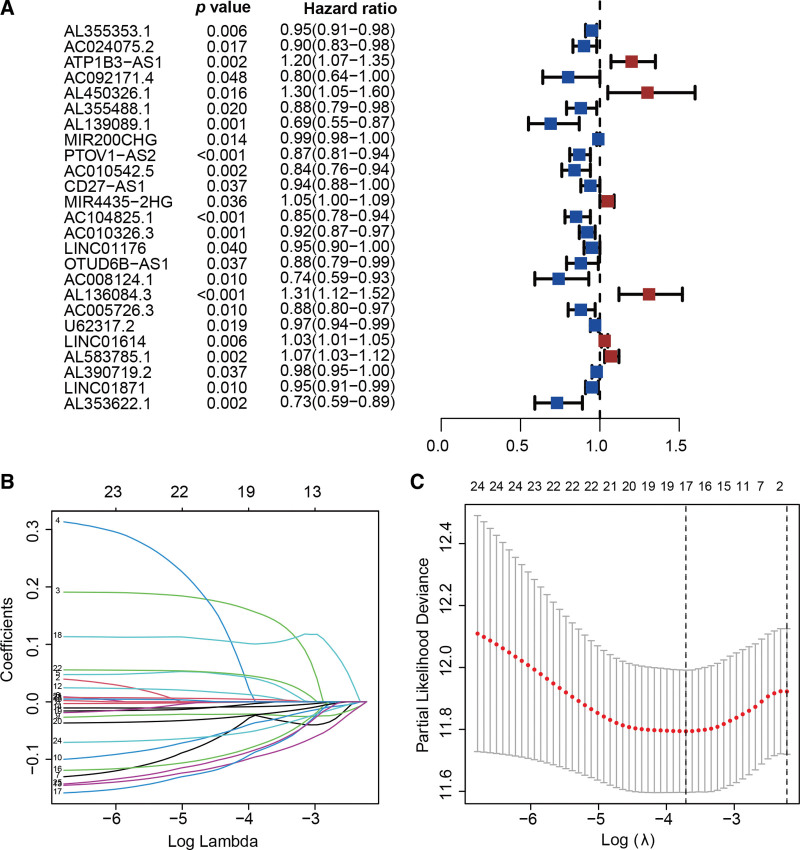
Identifying pyroptosis-related lncRNAs associated with OS. (A) The forest plot recognized 25 pyroptosis-related lncRNAs substantially associated with OS. (B) 17 lncRNAs were unveiled by the LASSO analysis to be associated with bladder cancer patients’ prognosis. (C) The LASSO coefficient spectrum for lncRNA associated with OS is presented. lncRNA = long noncoding RNA, LASSO = Least absolute shrinkage and selection operator, OS = overall survival.

**Figure 4.**
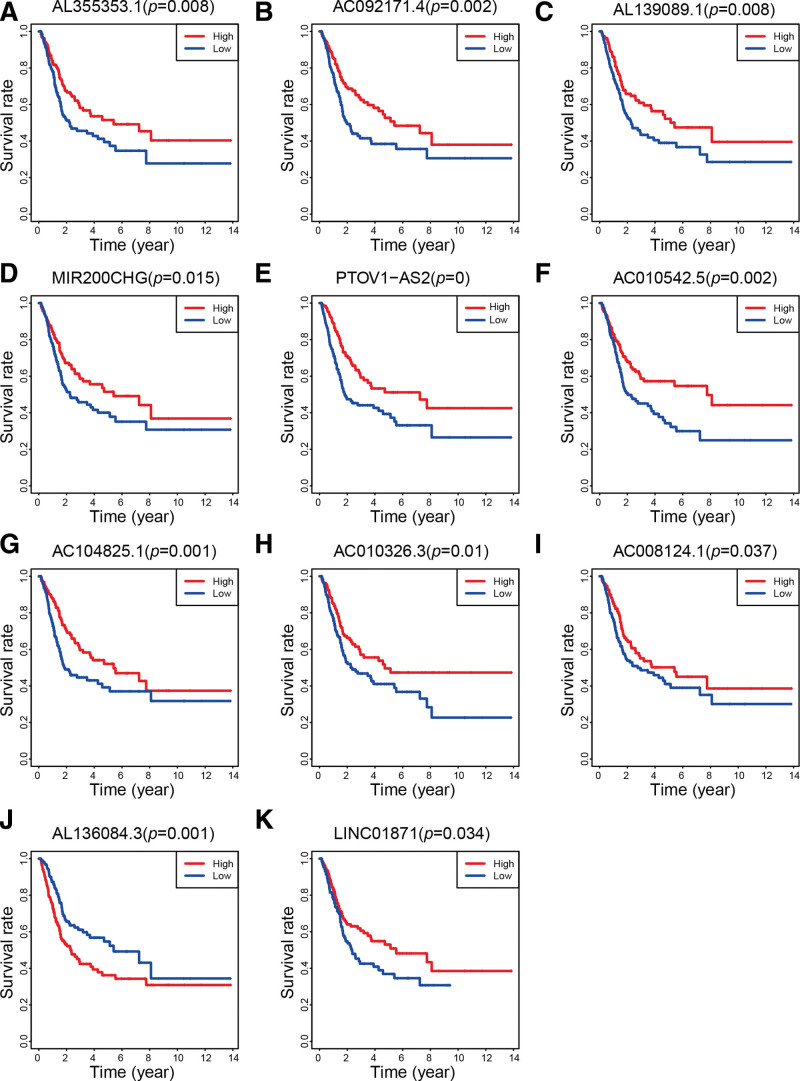
Survival analyses of lncRNAs associated with overall survival. Kaplan–Meier curves for (A) AL355353.1, (B) AC092171.4, (C) AL139089.1, (D) MIR200CHG, (E) PTOV1-AS2, (F) AC010542.5, (G) AC104825.1, (H) AC010326.3, (I) AC008124.1, (J) AL136084.3, and (K) LINC01871. lncRNA = long noncoding RNA.

Figure 1 presented the flowchart of our investigation. The TCGA database was utilized for gathering LncRNA sequencing data and clinical information. Finally, a total of 411 tumors and 19 adjacent normal tissues from 411 bladder cancer patients were extracted. From earlier studies, 52 pyroptosis-related genes were extracted. Four hundred and fifty-four lncRNAs were differentially expressed in tumors and adjacent normal tissues, including 161 up-regulated and 293 down-regulated in tumors (Fig. 2A). A total of 15 pyroptosis-related genes were screened to be differentially expressed, with 7 being up-regulated in tumors and 8 being down-regulated in tumors (Fig. 2B). Furthermore, using Pearson correlation analysis, we were successful in finding 59 pyroptosis-related lncRNAs. According to the univariate Cox regression analysis, 25 lncRNAs were related to prognosis (Fig. 3A). Then, we identified 17 lncRNAs by least absolute shrinkage and selection operator regression analysis (Fig. 3B and C). The Kaplan–Meier method thereby screened 11 lncRNAs tightly linked to OS of bladder cancer patients (Fig. 4).

### 3.2. Risk score model establishment

**Figure 5.**
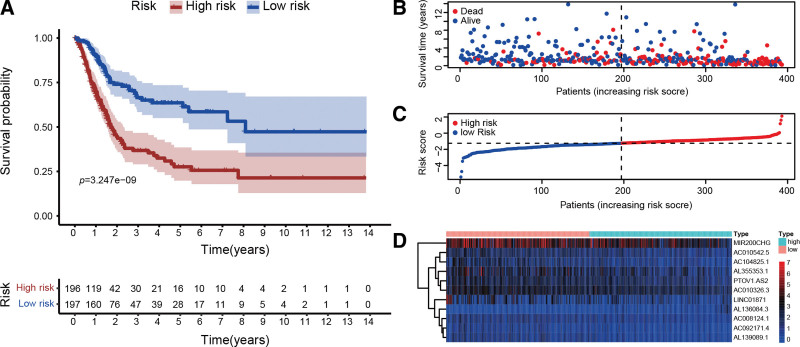
Risk score model establishment utilizing pyroptosis-related lncRNAs. (A) The Kaplan–Meier analysis unveiled a significantly shorter overall survival of the high-risk group than that of the low-risk group. (B) Each patient’s survival status. (C) Patient risk rating distributions. (D) Pyroptosis-related lncRNA heatmaps in low and high risk groups. Warm colors indicated a high level of expression, whereas cool colors indicated a low level of expression. lncRNA = long noncoding RNA.

The following is a risk score model that includes expression levels and βi of lncRNAs: Risk score = , × Ex). Patients were split into 2 groups, a high-risk group and a low-risk group, according to the median risk score. The high risk group’s OS was considerably shorter in comparison to the low risk group (*P* < .001, Fig. 5A). Compared to the risk coefficient and the mortality of patients of the low risk group, those of the high risk group were higher (Fig. 5B and C). From Figure 5D, the heatmap displayed the expression patterns of 11-lncRNA signatures of the 2 groups.

### 3.3. Nomogram construction and evaluation

**Figure 6.**
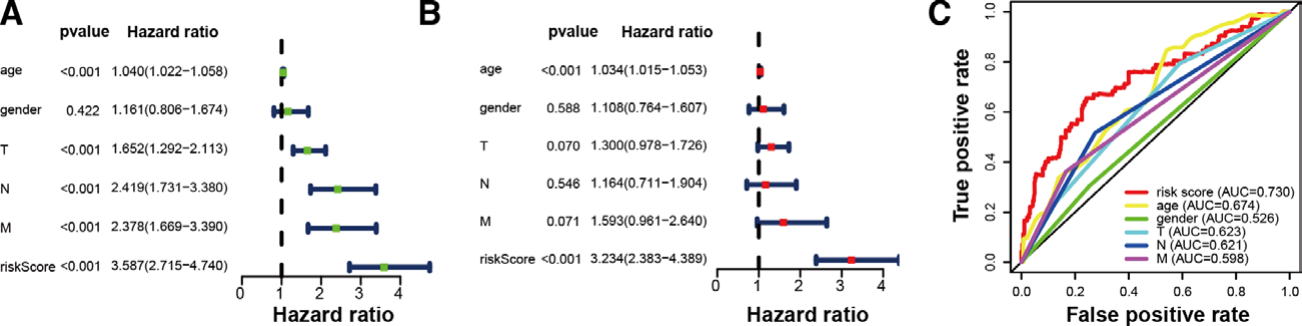
Analyses of prognostic values using the risk score and clinical variables. (A) The risk score was revealed to be strongly associated with overall survival in the univariate Cox analysis. (B) The 11-lncRNAs signature was identified to be an independent prognostic factor via the multivariate Cox analysis. (C) The 11-lncRNAs signature was an excellent prognostic predictor with an AUC of 0.730, according to receiver operating characteristic curve analysis. AUC = Area under the curve, lncRNA = long noncoding RNA.

**Figure 7.**
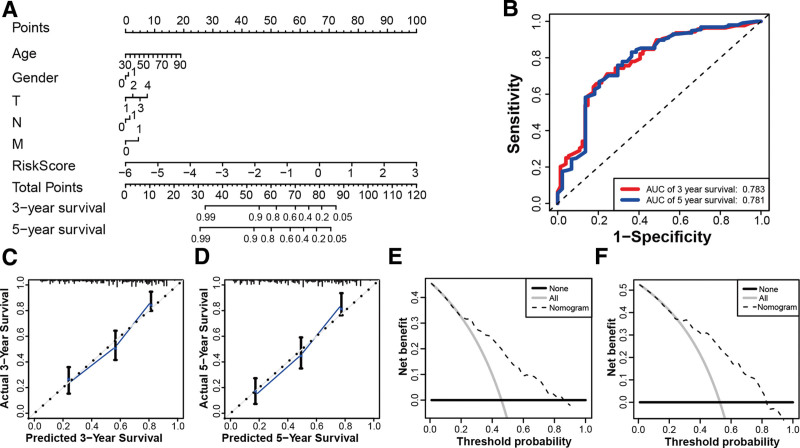
Establishment and interpretation of the nomogram for clinical variables and pyroptosis-related lncRNAs. (A) Establishment of the nomogram. (B) AUCs for 3- and 5-year OS were 0.783 and 0.781, respectively, according to ROC curve analysis. (C) The nomogram 3-year OS calibration curve. (D) The nomogram calibration curve for a 5-year OS. (E) The DCA for the nomogram 3-year OS. (F) The DCA for the nomogram 5-year OS. DCA = decision curve analysis, lncRNA = long noncoding RNA, OS = overall survival, ROC = receiver operating characteristic.

It was observed that the clinical variables and risk score were closely associated with OS in the univariate Cox regression (Fig. 6A). From Figure 6B, the 11-lncRNAs signature was also found to be an independent prognostic predictor for OS via the multivariate Cox analysis (*P* < .001). In addition, the receiver operating characteristic curve illustrated that the 11-lncRNAs signature was a remarkable prognostic predictor (AUC = 0.730, Fig. 6C). Then, using the multivariate Cox regression results, including clinical factors and risk scores, we constructed a nomogram (Fig. 7A). The nomogram AUCs for 3-year and 5-year OS were 0.783 and 0.781 respectively, as shown in Figure 7B. The nomogram concordance index of 0.86 indicated an excellent predictive effect. Besides, the calibration curve and decision curve analysis suggested that the model was valuable in predicting the prognosis (Figs. 7C–F).

### 3.4. Gene set enrichment analysis

**Figure 8.**
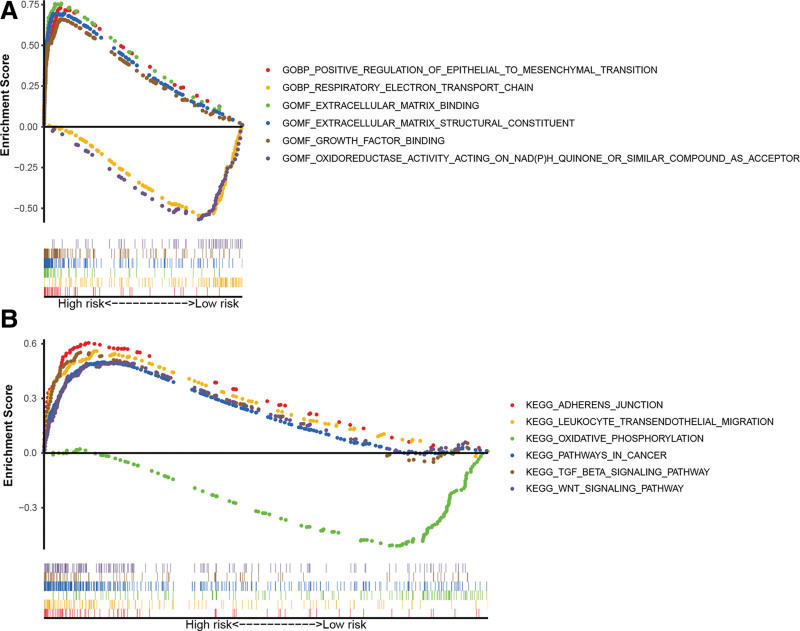
The results of Gene Set Enrichment Analysis. (A) According to the Gene Ontology enrichment analysis, pyroptosis-related lncRNAs were enriched in growth factor binding, oxidative stress, epithelial-mesenchymal transition, respiratory electron-transport chain, extracellular matrix binding, and structural constituent. (B) The Kyoto Encyclopedia of Genes and Genomes pathway analysis showed that these lncRNAs were relevant to cell adhesion, cancer pathway, oxidative phosphorylation, leukocyte transendothelial migration, TGF-β, and WNT signaling pathway. lncRNA = long noncoding RNA.

gene ontology enrichment analysis showed that the pyroptosis-related lncRNAs were enriched in growth factor binding, oxidative stress, epithelial-mesenchymal transition (EMT), respiratory electron-transport chain, extracellular matrix binding, and structural constituent (Fig. 8A). These lncRNAs were shown to be involved in cell adhesion, cancer pathway, oxidative phosphorylation, leukocyte transendothelial migration, TGF-β, and Wingless and INT-1 (WNT) signaling pathway by kyoto encyclopedia of genes and genomes pathway analysis (Fig. 8B). These elements may stimulate researchers to further investigate the mechanism of pyroptosis-related lncRNAs in bladder cancer pathogenesis.

## 4. Discussion

This study explored pyroptosis-related lncRNAs role in bladder cancer patients. The 11-lncRNAs signature was utilized to develop a risk model that separated patients with bladder cancer into a high-risk group and a low-risk group. Prognosis analysis revealed that the low-risk group patients presented favorable OS. Additionally, a nomogram was established and demonstrated to have a strong prognostic effect.

Among the lncRNAs, AL355353.1, AC092171.4, AL139089.1, MIR200CHG, PTOV1-AS2, AC010542.5, AC104825.1, AC010326.3, AC008124.1 and LINC01871 had a protective effect for prognosis, while AL136084.3 presented the opposite effect. AL136084.3 was linked to ferroptosis in bladder cancer and was found to be a prognostic risk factor.^[22,23]^ Meanwhile, Liu et al^[24]^ uncovered that PTOV1-AS2 was a good prognostic indicator for pancreatic cancer. However, further studies were needed to elucidate both biological and molecular mechanisms of these lncRNAs in bladder cancer.

Based on 11 pyroptosis-related lncRNAs, we established a relatively effective prediction model whose 3- and 5-year OS AUCs were 0.783 and 0.781, respectively. Several prognostic signatures for bladder cancer were identified in previous studies. Regarding the prognosis prediction for bladder cancer patients, Lai et al^[25]^ constructed and validated a predictive signature that included 9 immune-related lncRNAs. It was also found that autophagy-related,^[26]^ EMT-related,^[27]^ and N6-methyladenosine-related^[28]^ lncRNA signatures were strongly associated with the prognosis. What is more, Chen et al^[17]^ extracted a unique gene signature of 8 pyroptosis-related genes that might be leveraged in the prognosis prediction for bladder cancer patients. Several studies have investigated the prognostic role of pyroptosis-related lncRNAs in bladder cancer, while the construction or validation of the nomogram was lacking,^[29,30]^ or the signature AUC was relatively low. As a result, the prognostic value of pyroptosis-related lncRNAs in bladder cancer remains to be studied.

GSEA revealed that these lncRNAs were involved in oxidative stress, EMT, respiratory electron-transport chain, cell adhesion, oxidative phosphorylation, TGF-β, and WNT signaling pathway. The TGF-β signaling pathway is an essential pathway for the emergence and development of tumors with dual effects. Tamura et al^[31]^ pointed out that TGF-β can inhibit pyroptosis in breast cancer. In nonsmall cell lung cancer, WNT/β-catenin signaling was proved to promote progression by pyroptosis-related lncRNA FOXD2-AS1,^[32]^ while in colorectal cancer, FOXD2-AS1 may facilitate the progression through the regulation of the EMT signaling pathway.^[33]^ Suppression of oxidative stress can increase NLRP3 inflammasome-mediated cardiomyocyte pyroptosis through the NF-κB-GSDMD pathway.^[34]^ In bladder cancer, the pyroptosis-related lncRNAs might manage the proliferation and progression through the regulation of oxidative stress, EMT, cell adhesion, TGF-β, and WNT signaling pathway. However, the precise mechanism needs to be further studied.

Our research has a few limitations. For starters, it is just a retrospective study based on lncRNA data and a few clinical variables from the TCGA database, which is lacking in some detailed clinical information. Second, the prognostic value and the biological functions of pyroptosis-related lncRNAs are not yet fully elucidated. Finally, further in vivo or in vitro experiments are needed to investigate the concrete mechanisms of the lncRNAs.

## 5. Conclusions

In summary, the 11-lncRNAs signature was established to predict prognosis for bladder cancer patients. GSEA revealed that pyroptosis-related lncRNAs might modulate bladder cancer through the regulation of oxidative stress, EMT, cell adhesion, TGF-β, and WNT signaling pathway. Our study developed a well-validated nomogram based on these lncRNAs, which might offer new insights into bladder cancer prognosis.

## Acknowledgments

We thank the researchers and patients who contributed data to the TCGA database.

## Author contributions

**Conceptualization:** Zhe Lin, Wenfeng Xu.

**Data curation:** Guoliang Hou.

**Formal analysis:** Zhenyu Wu, Jie Zeng.

**Writing – original draft:** Zhenyu Wu, Jie Zeng.

**Writing – review & editing:** Mengxi Wu, Quan Liang, Bin Li, Zhe Lin, Wenfeng Xu.
